# A Paternal Fish Oil Diet Preconception Modulates the Gut Microbiome and Attenuates Necrotizing Enterocolitis in Neonatal Mice

**DOI:** 10.3390/md20060390

**Published:** 2022-06-13

**Authors:** Jelonia T. Rumph, Victoria R. Stephens, Sharareh Ameli, Philip N. Gaines, Kevin G. Osteen, Kaylon L. Bruner-Tran, Pius N. Nde

**Affiliations:** 1 Department of Microbiology, Immunology, and Physiology, Meharry Medical College, Nashville, TN 37208, USA; jrumph19@email.mmc.edu; 2 Women’s Reproductive Health Research Center, Department of Obstetrics and Gynecology, Vanderbilt University School of Medicine, Nashville, TN 37232, USA; victoria.r.stephens@vanderbilt.edu (V.R.S.); s.ameli@vanderbilt.edu (S.A.); philip.gaines@vumc.org (P.N.G.); kevin.osteen@vanderbilt.edu (K.G.O.); 3 The Vanderbilt Microbiome Initiative, Vanderbilt Microbiome Innovation Center, Vanderbilt University, Nashville, TN 37208, USA; 4 Department of Pharmacology, Vanderbilt University, Nashville, TN 37208, USA; 5 Department of Pathology, Microbiology, and Immunology, Vanderbilt University School of Medicine, Nashville, TN 37208, USA; 6 VA Tennessee Valley Healthcare System, Nashville, TN 37208, USA

**Keywords:** microbiome, nutrition, toxicants, multigenerational, developmental disease, therapeutic, necrotizing enterocolitis, formula, fish oil

## Abstract

Epidemiology and animal studies suggest that a paternal history of toxicant exposure contributes to the developmental origins of health and disease. Using a mouse model, our laboratory previously reported that a paternal history of in utero exposure to 2,3,7,8-tetrachlorodibenzo-p-dioxin (TCDD) increased his offspring’s risk of developing necrotizing enterocolitis (NEC). Additionally, our group and others have found that formula supplementation also increases the risk of NEC in both humans and mice. Our murine studies revealed that intervening with a paternal fish oil diet preconception eliminated the TCDD-associated outcomes that are risk factors for NEC (e.g., intrauterine growth restriction, delayed postnatal growth, and preterm birth). However, the efficacy of a paternal fish oil diet in eliminating the risk of disease development in his offspring was not investigated. Herein, reproductive-age male mice exposed to TCDD in utero were weaned to a standard or fish oil diet for one full cycle of spermatogenesis, then mated to age-matched unexposed females. Their offspring were randomized to a strict maternal milk diet or a supplemental formula diet from postnatal days 7–10. Offspring colon contents and intestines were collected to determine the onset of gut dysbiosis and NEC. We found that a paternal fish oil diet preconception reduced his offspring’s risk of toxicant-driven NEC, which was associated with a decrease in the relative abundance of the Firmicutes phylum, but an increase in the relative abundance of the Negativicutes class.

## 1. Introduction

The Developmental Origins of Health and Disease (DOHaD) concept suggests that the intrauterine environment has a significant impact on adult health. In the past, maternal factors were the target of many DOHaD studies, with only a few investigating the role of the father. This may be because the father’s sole contribution to pregnancy is his seminal fluid, which historically had not been theorized to play a large role in neonatal health. Recently, we have discovered that seminal fluid contributes to DOHaD, which means that paternal preconception diet and habits may influence the outcomes of not only his partner’s pregnancy but also their offspring’s postnatal health. 

Seminal fluid contains spermatozoa, nutrients, and microbes [1]. Our understanding of the transfer of sexually transmitted infections from male to female suggests a role for semen in influencing the vaginal and intrauterine microbiome [1]. Mandar et al. reported an association between males with genital tract inflammation and the predominance of *Gardnerella vaginalis* in their partners [2]; a bacterium that has been linked to poor pregnancy outcomes (e.g., preterm birth and chorioamnionitis) and has the potential to promote the onset of diseases of prematurity [3]. Seminal fluid has also been reported to influence conditions such as preeclampsia through the induction of immune tolerance in pregnant partners [1]. Together, these studies suggest that, in theory, mixing of the endometrial and seminal microbiome during conception could influence maternal–embryonic interactions during implantation and placentation, ultimately impacting offspring development [4].

Our laboratory previously reported that a history of in utero toxicant exposure reduced sperm quality in male mice and contributed to DOHaD in his offspring through placental dysfunction in his unexposed partner. This placental dysfunction translated to an increased risk of preterm birth and intrauterine growth restriction among their offspring [5]. We also reported that these offspring were susceptible to necrotizing enterocolitis (NEC), an inflammatory intestinal disease characterized by gut dysbiosis that impacts approximately 1 to 3 of every 1000 live births in North America. NEC is characterized by two forms of gut bacterial dysbiosis: Firmicute and Proteobacteria dysbiosis [6,7]. Infants with Firmicute dysbiosis typically develop NEC earlier than infants who experience Proteobacteria dysbiosis. Recent studies also suggest that infants susceptible to NEC present with increased abundances of Gammaproteobacteria, but decreased abundances of Negativicutes [8]. However, unlike Proteobacteria dysbiosis, Firmicute dysbiosis also affects approximately 10% of healthy infants, who do not develop NEC [7]. Hence, it is of importance to identify differences in the presentation of Firmicute dysbiosis in healthy infants versus those who are known to be susceptible to NEC. Additionally, it has been reported that a neonatal diet (e.g., formula feeding vs. maternal milk) also impacts the onset of gut dysbiosis in neonates [9,10]. It should also be noted that formula feeding is a risk factor for NEC in neonates [11]. 

Our group reported that intervening with a fish oil diet preconception in males with a history of toxicant exposure prevented preterm birth and intrauterine growth restriction in their offspring in association with improved placental function [12]. Neonatal development of offspring was also enhanced [13]. We hypothesized that this intervention would also reduce the risk of NEC since preterm birth and intrauterine growth restriction are independent risk factors for this disease. Therefore, in the present study, we aimed to determine if a paternal fish oil diet preconception influences early-life microbial seeding in his offspring and if this difference in microbial seeding is associated with a reduced risk of toxicant-driven NEC. We also examined the effect of postnatal formula supplementation since this diet has been linked to gut dysbiosis and the onset of NEC in human infants. 

We report that a paternal fish oil diet preconception altered Firmicute abundance in the gut of his offspring. A preconception fish oil diet increased the relative abundance of Negativicutes, a class of Firmicutes that are typically reduced in human infants with NEC [14]. Additionally, a preconception fish oil diet improved intestinal histology and reduced the risk of toxicant-driven NEC in offspring. To our understanding, we are the first to report a potential relationship between a father’s preconception diet, early-life microbial seeding, and the risk of NEC in his offspring.

## 2. Results

### 2.1. Paternal Preconception Diet Influences His Offspring’s Gut Microbial Diversity at the Phylum Level in F2_CT_ Pups

A major goal of our study was to determine whether paternal preconception diet or offspring postnatal diet influences bacterial diversity in the intestines of neonatal mice. Male C57BL/6 pups were weaned to a standard or a fish oil-supplemented diet for 7 weeks, then mated to age-matched C57BL/6 females maintained on the standard-diet preconception, during gestation, and postpartum. Offspring remained with the dam and were randomized to a strict maternal milk diet or a supplemental formula diet from postnatal day (PND) 7 through 10. On PND 11, the colon contents of offspring were collected and processed using Next-Generation Sequencing; pup nomenclature can be found in Table 1.

**Figure 1:**
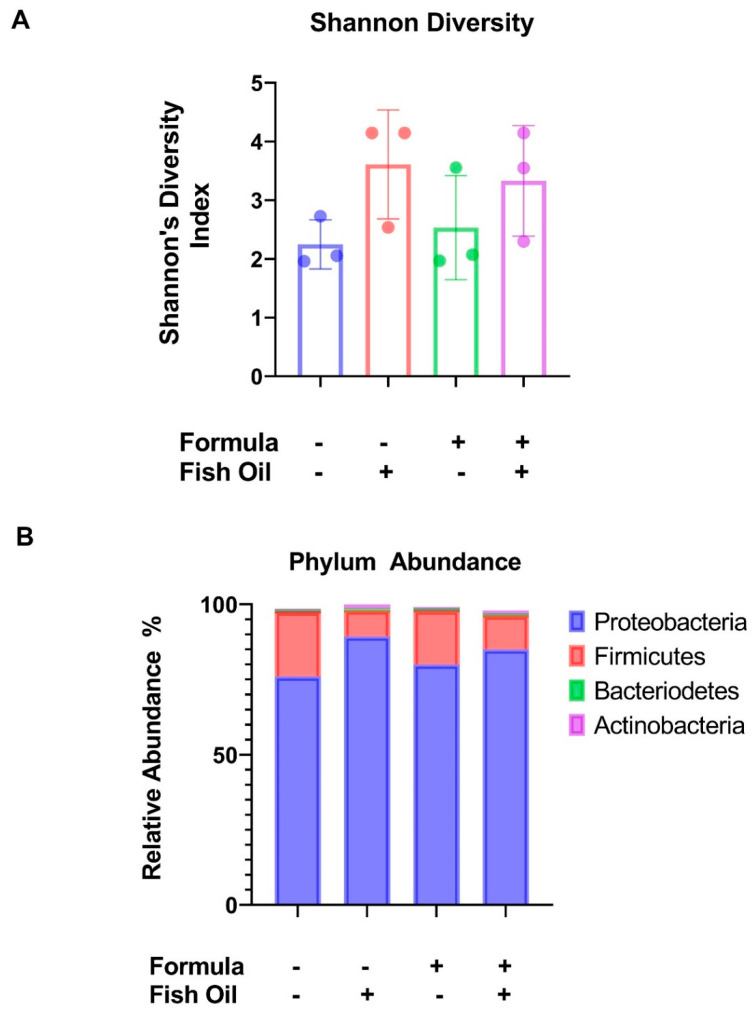
The effect of a preconception paternal fish oil diet and neonatal formula supplementation on intestinal microbial diversity in F2_CT_ pups: (**A**) Shannon Diversity analysis of maternal milk-fed or formula-supplemented pups sired by a father who received fish oil or standard diet preconception. (**B**) Contingency graph representing the abundance of different phyla in the colon content of offspring. Data represented as the average values of 3 non-littermate pups from each group.

When compared to maternal milk-fed pups sired by a father who received a standard diet (F2_CT_ pups), we found their formula supplemented counterparts, sired by standard diet fathers (F2_CT_/Form pups), exhibited similar Shannon Diversity Indexes (*p* = 0.9498). Compared to F2_CT_ pups, a paternal fish oil diet preconception led to a non-significant increase in the Shannon Diversity Index of maternal milk-fed pups (F2_CT_/Fish pups) (*p* = 0.1760) and formula supplemented offspring (F2_CT_/Fish/Form pups) (*p* = 0.3136) (Figure 1A). F2_CT_/Fish pups also exhibited a non-significant increase in the relative abundance of Proteobacteria (*p* = 0.2706), like the F2_CT_/Fish/Form pups (*p* = 0.5708). F2_CT_/Fish pups also displayed a small decrease in the relative abundance of Firmicutes (*p* = 0.2889), like the F2Fish/Form pups (*p* = 0.4658), but these trends did not reach significance (Figure 1B). 

### 2.2. Paternal Diet Preconception Influences the Relative Abundance of Firmicute Classes in F2_CT_ Pups

**Figure 2:**
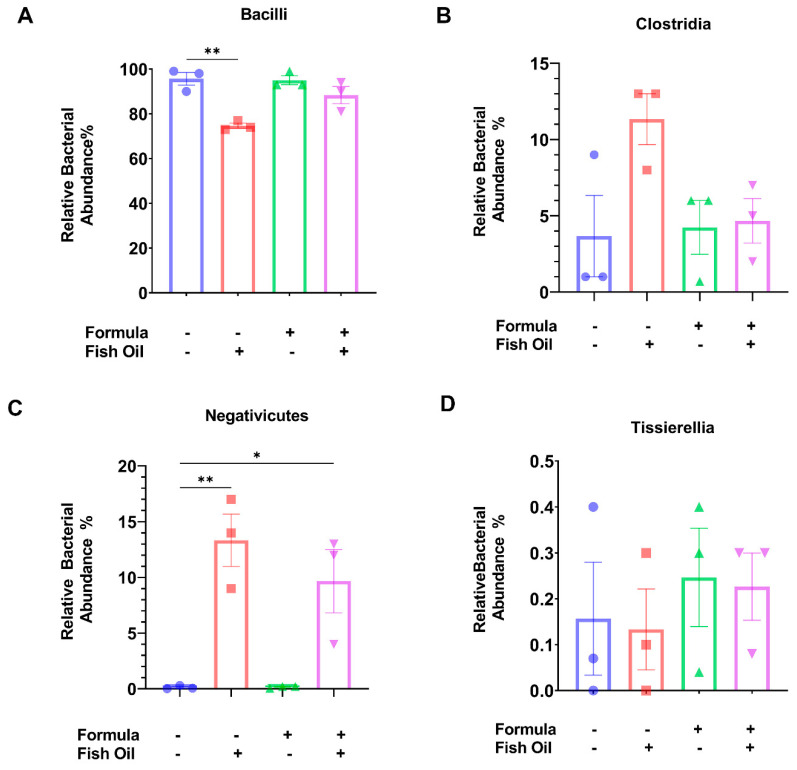
A paternal fish oil diet preconception affects the intestinal composition of Bacilli and Negativicutes in F2_CT_ pups: (**A**) Bar graphs represent the relative abundance of Bacilli, (**B**) Clostridia, (**C**) Negativicutes, and (**D**) Tissierellia in the colon content of offspring. Data were analyzed using one-way ANOVA and the Tukey Post Hoc Test; Data represent the mean ± SD from 3 non-littermates; * *p* ≤ 0.05, ** *p* ≤ 0.01.

Firmicute dysbiosis is suggested to be a biomarker of necrotizing enterocolitis (NEC) [6]; therefore, we examined how paternal and neonatal diet influenced classes of bacteria within the Firmicute phylum (i.e., Bacilli, Clostridia, Negativicutes, and Tissierellia). The relative abundance of Bacilli was similar between F2_CT_ and F2_CT_/Form pups (*p* = 0.9958). F2_CT_/Fish pups displayed a significant decrease in the relative abundance of Bacilli (*p* = 0.0014); however, F2_CT/_Fish/Form pups exhibited similar relative abundances of Bacilli (*p* = 0.1968) when compared to F2_CT_ pups (Figure 2A). F2_CT_/Fish pups also displayed a small increase in the relative abundance of Clostridia (*p* = 0.0573). F2_CT_/Fish/Form pups exhibited a relative abundance of Clostridia that was like the F2_CT_ pups (*p* = 0.9670) (Figure 2B). Significantly, the relative abundance of Negativicutes was increased in F2_CT_/Fish (*p* = 0.0025) and F2_CT_/Fish/Form offspring (*p* = 0.0160) (Figure 2C). There was no difference in the relative abundance of Tissierellia between groups (Figure 2D). 

### 2.3. Paternal Diet Preconception Influences the Relative Abundance of Bacilli and Negativicutes Orders in F2_CT_ Pups

**Figure 3:**
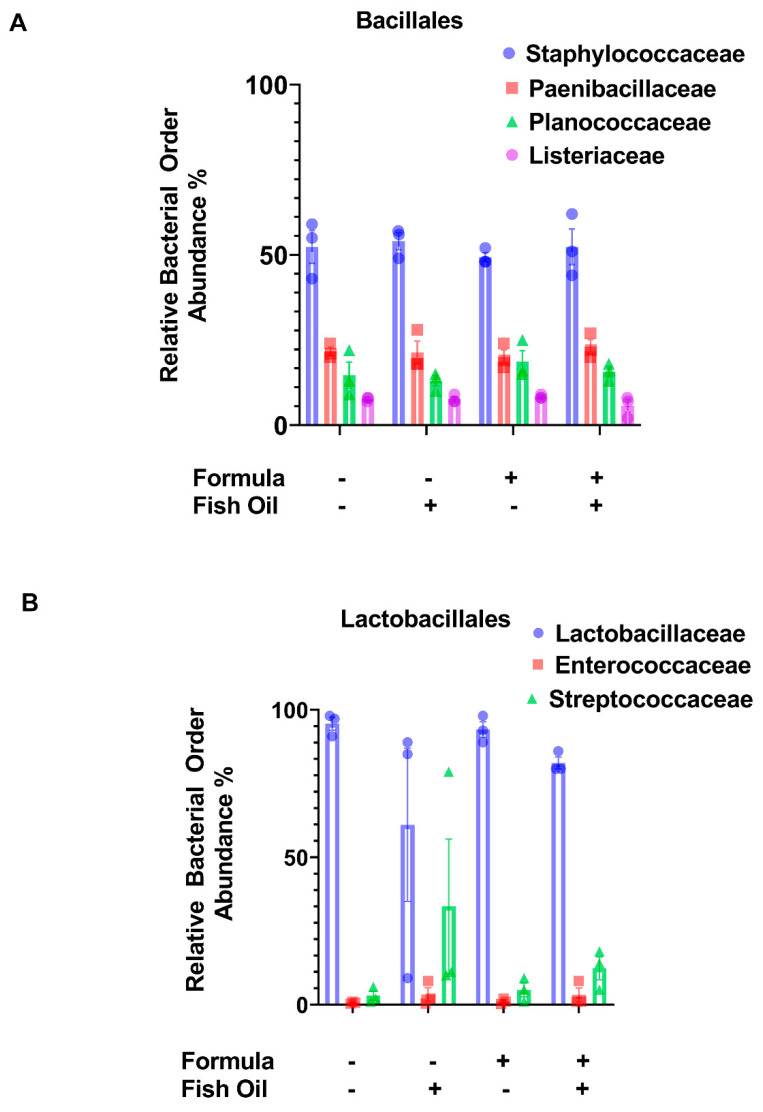
A paternal fish oil diet preconception does not impact bacterial groups classified as Bacilli in the intestines of F2_CT_ pups. Bar graphs represent the relative abundances of bacterial groups within (**A**) Bacillales and (**B**) Lactobacillales in the colon content of offspring. Data represent the mean ± SD from 3 non-littermates.

**Figure 4:**
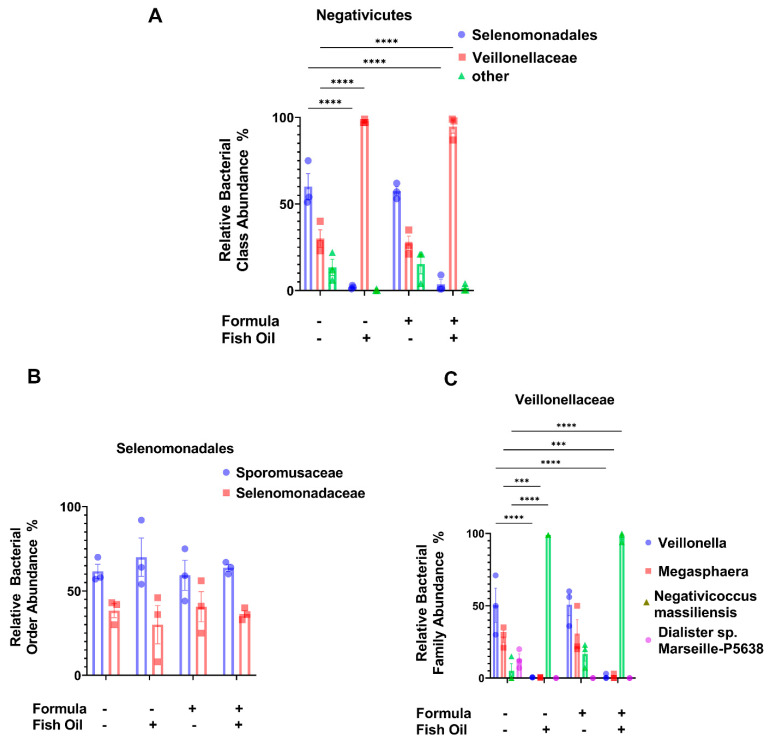
A paternal fish oil diet preconception influences Negativicutes orders in the intestines of F2_CT_ pups: (**A**) Bar graphs represent the relative abundance of Negativicutes, (**B**) *Veillonellaceae*, and (**C**) Selenomondales in the colon content of offspring. Data analyzed using one-way ANOVA and the Tukey Post Hoc Test; Data represent the mean ± SD from 3 non-littermates *** *p* ≤ 0.001, **** *p* ≤ 0.0001.

Since a paternal fish oil diet led to a significant decrease in the relative abundance of Bacilli, we measured the relative abundances of bacterial groups within this class. We found that a paternal fish oil diet preconception and/or neonatal formula supplementation did not have a significant impact on the relative abundance of bacterial groups within the orders of Bacillales (*p* ≥ 0.5869) and Lactobacillales (*p* ≥ 0.0652) (Figure 3A,B). Since a paternal fish oil diet led to a significant increase in the relative abundance of Negativicutes (Figure 2C), herein, we also measured the relative abundances of bacterial groups within this class. We found that compared to pups of fathers maintained on the standard diet, a paternal fish oil diet preconception was associated with a significant decrease in the relative abundance of the order Selenomondales in F2_CT_/Fish and F2_CT_/Fish/Form pups (*p* < 0.0001). The relative abundance of the *Veillonellaceae* family was also significantly decreased in F2_CT_/Fish (*p* < 0.0001) and F2_CT_/Fish/Form offspring (*p* < 0.0001) compared to F2_CT_ offspring (Figure 4A).

We further investigated how paternal and neonatal diets influenced the relative abundances of bacterial groups classified under the order of Selenomondales and the family of *Veillonellaceae*. We found that paternal and neonatal diets did not greatly impact the relative abundances of *Sporomusaceae* (*p* ≥ 0.7805) or *Selenomonadaceae* (*p* ≥ 0.7805) (Figure 4B). We also report that, compared to F2_CT_ pups, F2_CT_/Fish and F2_CT_/Fish/Form pups displayed significant reductions in the relative abundances of *Veillonella* (*p* < 0.0001) and *Megasphaera* (*p* ≥ 0.0007). F2_CT_/Fish and F2_CT_/Fish/Form pups also displayed a significant increase in the relative abundance of *Negativicoccus massiliensis* (*p* < 0.0001) compared to F2_CT_ pups. F2_CT_/Form pups displayed no significant difference in the relative abundances of *Veillonella*, *Megasphaera*, or *Negativicoccus massiliensis* (*p* ≥ 0.1607) (Figure 4C) compared to F2_CT_ pups. 

### 2.4. Paternal Diet Preconception Alters the Firmicute Abundance in F2_TCDD_ Pups

As described above, we observed that a paternal fish oil diet preconception was associated with a small increase in offspring Shannon Diversity Index as well as an altered relative abundance of Negativicutes and associated bacterial groups. An increased Shannon Diversity Index and an increase in the presence of Negativicutes are associated with a reduced risk of developing NEC in human infants [6,8,15,16]. Therefore, our next goal was to employ our toxicant-driven murine model of NEC to determine if intervening with a paternal fish oil diet preconception improves NEC outcomes by influencing microbial diversity in offspring. In this model, the offspring of males with a history of in utero 2,3,7,8-tetrachlorodibenzo-p-dioxin (TCDD) exposure are susceptible to NEC and postnatal formula supplementation further increases their risk of disease [17]. 

**Figure 5:**
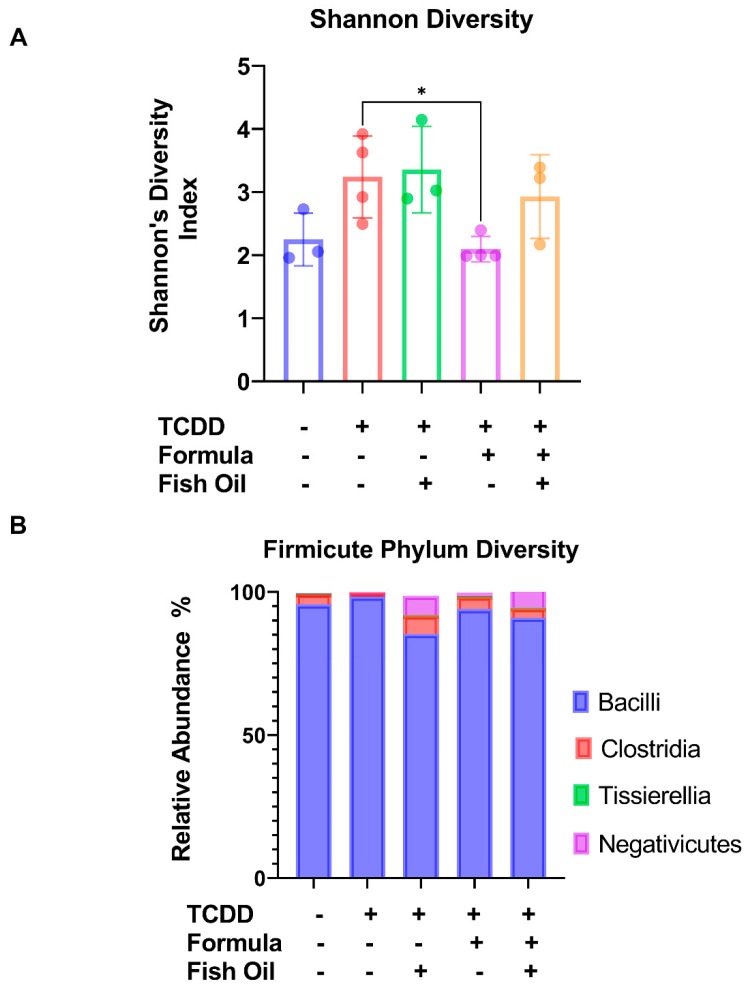
A paternal fish oil diet alters Firmicute diversity in the intestines of F2_TCDD_ pups: (**A**) Shannon Diversity analysis of maternal milk-fed or formula-supplemented F2_TCDD_ pups sired by a father that received fish oil or a standard diet preconception. (**B**) Contingency graph representing the abundance of different phyla in the colon content of offspring. Data were analyzed using one-way ANOVA and the Tukey Post Hoc Test; Data represent the mean ± SD from 3 non-littermates; * *p* ≤ 0.05.

We report no significant difference in the Shannon Diversity index of maternal milk-fed pups sired by standard diet fathers with a history of TCDD exposure (F2_TCDD_ pups) when compared to F2_CT_ pups. However, formula supplemented pups sired by standard diet fathers with a history of TCDD exposure (F2_TCDD_/Form pups) exhibited a significant decrease in their Shannon Diversity Index compared to F2_TCDD_ pups (*p* = 0.0384) (Figure 5A). Next, we observed the relative abundance of Firmicute classes among pups with a history of TCDD exposure. When compared to F2_CT_ pups, maternal milk fed pups sired by fathers with a history of TCDD exposure who received fish oil (F2_TCDD_/Fish pups) displayed a significant decrease in their relative abundance of Bacilli (*p* = 0.0002). Additionally, F2_TCDD_/Fish pups exhibited a significant increase in their relative abundance of Negativicutes compared to F2_CT_ pups (*p* = 0.0205). These trends persisted in formula supplemented pups sired by fathers with a history of TCDD exposure who received fish oil (F2_TCDD_/Fish/Form pups), but did not reach significance. We report no significant differences in the relative abundances of Clostridia and Tissierellia between groups. However, F2_TCDD_/Fish pups (*p* < 0.0001), and F2_TCDD_/Fish/Form pups (*p* = 0.0082) displayed a significant reduction in the relative abundance of Bacilli compared to F2_TCDD_ pups. F2_TCDD_/Fish pups also exhibited a significant increase in the relative abundance of Negativicutes when compared to F2_TCDD_ pups (*p* = 0.0185). However, this trend did not reach significance in F2_TCDD_/Fish/Form pups (*p* = 0.0543) (Figure 5B).

### 2.5. Paternal Fish Oil Consumption Alters the Abundance of Negativicutes in F2_TCDD_ Pups

**Figure 6:**
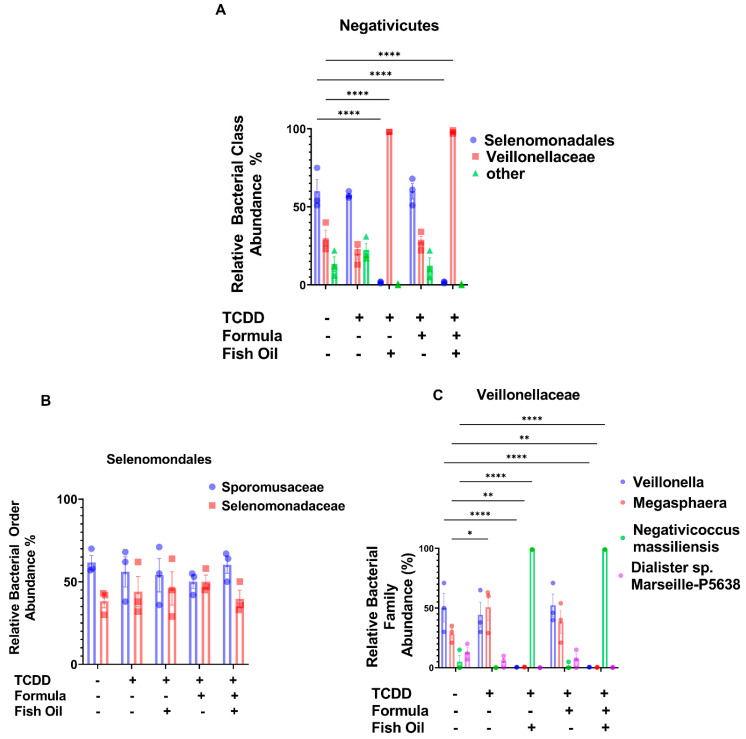
A paternal fish oil diet preconception influences Negativicutes orders in the intestines of F2_TCDD_ pups: (**A**) Bar graphs represent the relative abundance of Negativicutes, (**B**) *Veillonellaceae,* and (**C**) Selenomondales in the colon content of offspring. Data analyzed using one-way ANOVA and the Tukey Post Hoc Test; Data represent the mean ± SD from 3 non-littermates; * *p* < 0.05, ** *p* < 0.01, **** *p* ≤ 0.0001.

Next, we examined whether a paternal fish oil diet also increased the relative abundance of bacterial groups within the class of Negativicutes in pups with a history of TCDD exposure. F2_TCDD_/Fish and F2_TCDD_/Fish/Form pups exhibited a significant increase in the relative abundances of Selenomondales and *Veillonellaceae* when compared to F2_CT_ pups (*p* < 0.0001) (Figure 6A). Like what was demonstrated by F2_CT_/Fish pups, a paternal fish oil diet preconception did not affect the relative abundances of *Sporomusacae* and *Selenomonadaceae* in pups with a history of TCDD exposure (Figure 6B). F2_TCDD_ pups exhibited a significant increase in the relative abundance of *Megaspahera* (*p* = 0.0302), but no differences in the relative abundance of *Veillonella*, *Negativicoccus massilienis* or *Dialister* sp. *Marseille-P5638* when compared to F2_CT_ pups. F2_TCDD_/Form pups exhibited similar abundances of *Veillonella*, *Megasphaera*, *Negativicoccus massilienisis,* and *Dialaister* sp. *Marseillie-P5638* when compared to F2_CT_ pups. F2_TCDD_/Fish and F2_TCDD_/Fish/Form pups exhibited a significant decrease in their relative abundance of *Veillonella* and *Megasphaera* (*p* < 0.0001), but a significant increase in *Negativicoccus massiliensis* (*p* < 0.0001) when compared to F2_CT_ pups (Figure 6C). Additionally, F2_TCDD_/Fish and F2_TCDD_/Fish/Form pups exhibited a significant decrease in the relative abundance of Selenomondales and *Veillonellaceae* when compared to F2_TCDD_ pups (*p* < 0.0001) (Supplementary Figure S1A). Significantly, F2_TCDD_/Fish and F2_TCDD_/Fish/Form pups displayed a decrease in *Veillonella* and *Megaspahera*, but an overall increase in *Negativicoccus massiliensis* (*p* < 0.0001) when compared to F2_TCDD_ pups (Supplementary Figure S1B). 

### 2.6. A Paternal Fish Oil Diet Preconception Attenuates Susceptibility to NEC

**Figure 7:**
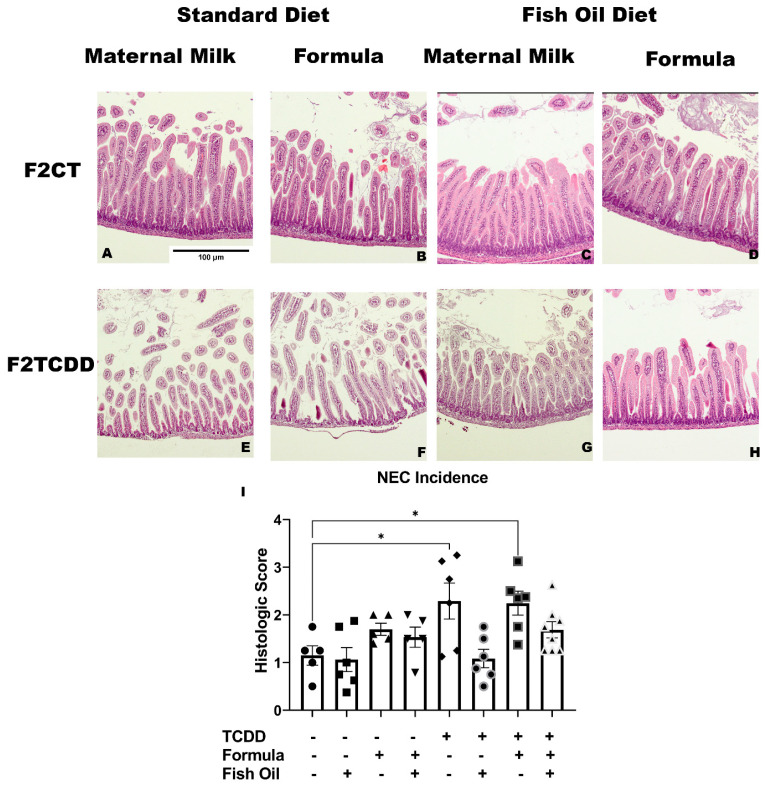
A paternal fish oil diet preconception improves intestinal histology and NEC outcomes among F2_CT_ and F2_TCDD_ pups: (**A**) Representative photomicrographs of the intestinal histology of F2_CT_ pups, (**B**) F2_CT_/Form pups, (**C**) F2_CT_/Fish pups, (**D**) F2_CT_/Fish/Form pups, (**E**) F2_TCDD_ pups, (**F**) F2_TCDD_/Form pups, (**G**) F2_TCDD_/Fish pups, (**H**) F2_TCDD_/Fish/Form pups, (**I**) NEC outcomes among all groups. Data were analyzed using one-way ANOVA and the Tukey Post Hoc Test; Data represent the mean ± SD from *n* ≥ 5 non-littermates; * *p* < 0.05.

Our final aim was to determine if a paternal fish oil diet preconception reduced the risk of NEC in his offspring. F2_CT_ and F2_CT_/Fish pups exhibited healthy intestines, denoted by elongated enterocytes, little-to-no sloughing of the intestinal crypt from the villi, and integrity of the submucosa (Figure 7A,C). Additionally, F2_CT_ and F2_CT_/Fish pups did not develop NEC (Figure 7B and Table 2). F2_CT_/Form pups displayed mild intestinal sloughing (Figure 7B) and 40% of these pups developed low-grade NEC (Figure 7I and Table 2). Limited intestinal sloughing was observed in F2_CT_/Fish/Form pups (Figure 7D) and the incidence of NEC was reduced to 20% in this group (Figure 7I and Table 2). 

F2_TCDD_ pups exhibited severe intestinal sloughing and impaired submucosa integrity compared to F2_CT_ pups and this trend persisted in F2_TCDD_/Form pups (Figure 7E,F). However, F2_TCDD_/Fish and F2_TCDD_/Fish/Form pups displayed improved intestinal integrity and enterocyte elongation. F2_TCDD_/Fish and F2_TCDD_/Fish/Form pups also exhibited a reduction in the separation of intestinal crypt and villi (Figure 7G,H). This improvement in intestinal histology among F2_TCDD_/Fish and F2_TCDD_/Fish/Form pups translated to an attenuated risk for the development of NEC. F2_TCDD_ pups (*p* = 0.0292) and F2_TCDD_/Form pups (*p* = 0.0407) had the highest incidences of NEC at 66% and 83%, but the risk of NEC was eliminated in F2_TCDD_/Fish pups and reduced to 58% in F2_TCDD_/Fish/Form pups (Figure 7I and Table 2). Overall, a paternal fish oil diet preconception reduced the risk of NEC in his offspring. Interestingly, offspring sired by fathers who received a fish oil diet preconception displayed similarities in their Firmicute abundances independent of the father’s history of TCDD exposure (Table 3). This suggests that Firmicute dysbiosis may have a similar phenotype in healthy versus unhealthy infants and the onset of NEC in infants with Firmicute dysbiosis is also driven by additional risk factors such as prematurity, formula supplementation, and inflammation. 

## 3. Discussion

Necrotizing enterocolitis (NEC) is a rare, but serious, intestinal inflammatory disease. Although NEC most commonly affects infants that were born preterm, up to 10% of babies with this condition are delivered at term [18]. Some groups have also reported that mortality rates associated with NEC are similar among term and preterm infants [19]. Both term and preterm infants can exhibit characteristics, such as Firmicute dysbiosis, which have been linked to NEC. Thus, it is important to identify factors that influence microbial seeding in early life and determine whether these factors influence the onset of NEC. Herein, our major goals were to determine the impact that paternal preconception diet and offspring postnatal diet had on early-life microbiome composition, determine whether a father’s history of toxicant exposure impacted his offspring’s intestinal microbiome, and, finally, determine if any of these parameters were associated with the onset of NEC. 

To determine whether paternal or neonatal diet influenced microbial diversity in F2_CT_ mice, we provided their fathers with a fish oil diet preconception. We previously reported that this diet influenced multiple sperm parameters, which positively impacted placental function, pregnancy outcomes, and offspring development [12]. Our findings, presented herein, demonstrate that this diet slightly increased microbial diversity in F2_CT_/Fish and F2_CT_/Fish/Form pups (Figure 1A). Furthermore, F2_CT_/Fish and F2Fish/Form pups displayed a reduction in their relative abundance of Firmicutes (Figure 1B). These data support the theory that priming of neonatal microbial seeding may begin in utero [20]. It should be noted that our findings are in alignment with a murine study conducted by Yu et al. suggesting that direct fish oil treatment decreased the relative abundance of the Firmicute phylum [21]. Additional studies from others have suggested that the omega-3 fatty acids found in fish oil can influence the gut microbiome by modulating the growth of opportunistic bacteria [22]. 

Next, we aimed to identify differences in the relative abundances of bacterial classes within the Firmicute phylum to observe characteristics of dysbiosis. We reported that a paternal fish oil diet preconception had a more significant impact on these parameters than a neonatal diet. F2_CT_/Fish pups displayed a significant reduction in their relative abundance of Bacilli (Figure 2A), but a non-significant increase in their relative abundances of Clostridia (Figure 2B). Additionally, F2_CT_/Fish and F2_CT_/Fish/Form pups exhibited a significant increase in their relative abundance of Negativicutes (Figure 2C); however, the relative abundance of Tissierellia was not impacted (Figure 2D). These findings confirm that fish oil can influence the relative abundance of Bacilli, a concept that was previously reported by Curone et al. using a rabbit model [23]. Unlike our findings, other groups have reported that fish oil reduces the abundance of Clostridia in humans and mice [24,25]—suggesting that this may be the case when fish oil is provided to the subject directly but not when the supplement is provided indirectly as in our study. 

Since a paternal fish oil diet led to a significant decrease in the relative abundance of Bacilli, we further investigated the relative abundances of Bacialles (Figure 3A) and Lactobacialles (Figure 3B) but found no differences between the groups. Likely because these bacterial groups are theorized to be transferred from mother to infant during birth and maternal feeding [26,27]. Others have reported that Bacilli abundance is increased following neonatal formula supplementation [28]; although, our data support this finding, the trend did not reach significance (Figure 2 and Figure 3A,B).

Our next aim was to investigate the relationship between paternal and neonatal diet and the relative abundance of Negativicutes in the gut of offspring. A study conducted by Hosomi et al. suggests that the dietary fish oil fatty acids eicosapentanoic acid and docosahexaenoic acid increased the relative abundance of Negativicutes in the intestines of rats who were provided the diet directly [29]. Supporting the study conducted by Hosomi et al., we report that F2_CT_/Fish and F2_CT_/Fish/Form pups had a significantly increased relative abundance of Negativicutes (Figure 2C), which was associated with a significant increase in the abundance of *Veillonellaceae* and a significant decrease in the abundance of Selenomondales (Figure 4A). Paternal and neonatal diet did not influence families within the order of Selenomondales (Figure 4B); however, F2_CT_/Fish and F2_CT_/Fish/Form pups exhibited reduced abundances of *Veillonella* and *Megashpaera* but increased abundances of *Negativicoccus massiliensis* (Figure 4C). To our knowledge, we are the first group to examine the relationship between the abundance of Negativicutes in offspring and paternal diet preconception. 

Our final aim was to determine if a paternal fish oil diet preconception could reduce the risk of his offspring developing toxicant-driven NEC in association with modulation of the gut microbiome. A reduction in microbial diversity, marked by a decrease in the Shannon Diversity Index is a characteristic of NEC [30] and we report that F2_TCDD_/Form pups exhibited a significant decrease in their Shannon Diversity Index compared to F2^TCDD^ pups (Figure 5A). This supports our previous report that formula supplementation increases the risk of NEC in pups with a history of toxicant exposure [17]. Additionally, F2_TCDD_ pups displayed an over-growth of Bacilli but F2_TCDD_/Fish pups displayed a reduction in their relative abundance of Bacilli, but an increase in the relative abundance of Negativicutes, respectively (Figure 5B).

It should be noted that the absence of Negativicutes in the intestines of neonates has been linked to the development of NEC in humans [8]. F2_TCDD_/Fish pups and F2_TCDD_/Fish/Form pups displayed a significant increase in their relative abundance of Negativicutes. Similar to F2_CT_/Fish pups, F2_TCDD_/Fish and F2_TCDD_/Fish/Form pups had a significantly increased relative abundance of *Veillonellaceae*, but a significantly decreased relative abundance of Selenomondales (Figure 6A). Additionally, the trend of a paternal fish oil diet having no impact on the relative abundance of bacterial members within Selenomondales persisted (Figure 6B). Additionally, the trend of a paternal fish oil diet decreasing the relative abundance of *Veillonella* and *Megaspahera*, while significantly increasing the relative abundance of *Negativicoccus massiliensis* also persisted in F2_TCDD_/Fish and F2_TCDD_/Fish/Form pups (Figure 6C). 

In this study, we confirmed our previous report that a history of paternal toxicant exposure and postnatal formula supplementation are independent risk factors for NEC [17]. Herein, 66% of F2_TCDD_ pups and 83% of F2_TCDD_/Form pups developed NEC; whereas 0% of F2_CT_ pups and 40% of F2_CT_/Form pups developed NEC. The risk of NEC was eliminated in F2_CT_/Fish pups and reduced to 20% in F2_CT_/Fish/Form pups. This trend persisted in pups with a history of toxicant exposure, as a fish oil intervention reduced their incidence of NEC to 0% in F2_TCDD_/Fish pups and 25% in F2_TCDD_/Fish/Form pups (Table 2). Additionally, F2_CT_/Form pups exhibited intestinal sloughing; however, this sloughing was more severe and accompanied by impaired intestinal integrity in the F2_TCDD_ and F2_TCDD_/Form pups (Figure 7A). Although some F2_CT_/Form pups developed NEC, these incidences did not reach significance, unlike F2_TCDD_ and F2_TCDD_/Form pups (Figure 7B). 

Overall, our data suggest that a paternal fish oil diet preconception may have a similar effect on microbial seeding in F2_CT_ neonatal mice and F2_TCDD_ neonatal mice (Table 3). Our findings also suggest that formula supplementation and a history of paternal TCDD exposure are independent risk factors for NEC in neonatal mice. A paternal fish oil diet eliminated the risk of NEC in maternal milk fed offspring with a history of toxicant exposure and reduced the risk of disease in formula supplemented neonatal mice. Our data suggest that a paternal fish oil diet preconception helps reduce the risk of NEC by modulating the neonatal gut microbiome; however, our findings also suggest that additional factors (e.g., prematurity, inflammation, and formula supplementation) drive NEC outcomes as well. 

We previously established that the mothers of pups sired by fathers with a history of TCDD exposure exhibit poor placental function, which put their offspring at risk of early-life complications. As has been demonstrated by us and others, the placenta is heavily influenced by the father and plays significant roles in fetal and neonatal development. We also reported that the mothers of F2_TCDD_/Fish pups had improved placental function and their offspring were less susceptible to early-life conditions, likely because this paternal dietary intervention improved sperm function, which translated to improved placental function [12]. Additionally, the placental phenotype has been suggested to contribute to the development of the infant gut microbiome as a consequence of fetal ingestion of amniotic fluid [31,32,33]. However, to our knowledge, ours is the first study to explore the neonatal microbiome following toxicant exposure of the father. Our study also suggests that the father’s preconception diet may also influence the microbial landscape of his offspring, potentially due to changes in placental function. However, it should be noted that the current study used a small sample size of three pups per group from microbial analyses; therefore, larger group sizes are needed to confirm the trends that we have reported. Additionally, due to the non-invasive nature of this study, it would also be worthwhile to measure these outcomes using an epidemiological study design.

## 4. Materials and Methods

### 4.1. Animals

Adult (10–12 weeks) and neonatal C57BL/6 mice were used in this study. Adult mice were obtained from Envigo (Indianapolis, IN, USA) or born in-house. All neonatal mice were born in-house. Animals were housed in the Barrier Animal Care Facility at Vanderbilt University Medical Center, which is free of common mouse pathogens. Adult mice were provided food and water ad libitum. Animal room temperatures were maintained between 22 and 24 °C and relative humidity of 40–50% on a 12 h light:dark schedule. Experiments described in this study were approved by Vanderbilt University’s Institutional Animal Care and Use Committee (IACUC) per the Animal Welfare Act protocol #M2000098.

### 4.2. Chemicals

TCDD (99% in nonane #ED-908) was purchased from Cambridge Isotope Laboratories (Andover, MA, USA). Esbilac Puppy Milk Replacer Powder was purchased from Pet-Ag, Inc (Hampshire, IL, USA). 

### 4.3. Mating, Exposure, and Diet Scheme

Virgin 10 to 12-week-old C57BL/6 females were mated with intact males of similar age. Females were weighed daily and monitored for the presence of a vaginal semen plug; denoting copulation had occurred. The morning a vaginal plug was identified, the F0 dam was considered pregnant (denoted as embryonic day 0.5 (E0.5)) and moved to a new cage. F1 offspring were typically born on E20 and weaned at 3 weeks. 

#### 4.3.1. TCDD Exposure

F0 dams were exposed to 10 µg/kg of TCDD in corn oil or corn oil vehicle by gavage on E15.5 as previously described [13,17]. 

#### 4.3.2. Diet and Mating Scheme for the F1 Generation

Purina Mills (TestDiet division) provided the 5% Menhaden fish oil diet, which also contained 1.5% corn oil to prevent depletion of omega-6 fatty acids. Menhaden fish oil, (OmegaProtein, Houston, TX, USA) has an established fatty acid profile (~40% omega-3 fatty acids) and was processed to remove dioxins and polychlorinated biphenyls. The fish oil diet is a modification of Purina’s low phytoestrogen rodent chow, 5VR5, which was used as the control (standard) diet. Both diets are matched for energy content, but the fish oil diet had increased fat content with slightly lower protein and carbohydrate content. The fish oil diet was maintained in vacuum-sealed bags at (−20 °C) until use and once provided to mice, replaced every 3 days.

F1 males were weaned for 7 weeks on a fish oil or standard diet (one full cycle of spermatogenesis) and mated at 10–12 weeks of age with age-matched unexposed C57BL/6 females. Once a vaginal semen plug was identified, dams were singly housed until parturition. F2 offspring were typically born between E18.5 and 20. 

### 4.4. Formula Feeding

As previously described [13,17], beginning on postnatal day 7 (PND7), pups were weighed, and randomized to a strict maternal milk diet or a supplemental formula diet. Pups were bottle-fed 30 µL of freshly prepared Esbilac formula diluted in autoclaved cage water three times a day over the course of four days using a small nipple attached to a 1 ml syringe (Miracle Nipple Mini for Pets and Wildlife). Each 30 µL dose was provided in two aliquots of 15 µL, each 10 min apart. All pups remained with dams for the duration of the study and were allowed to nurse ad libitum. 

### 4.5. Euthanasia and Sample Collection

On PND11 at 1100 h local time, pups were weighed, then euthanized by decapitation performed under deep anesthesia per AAALAC guidelines. Following euthanasia, the peritoneal cavity was opened, and the intestines were identified. The colon was then excised from the small intestine and rectum. Upon removal of the colon, sterile tools were used to push the colon contents out of the colon and into a sterile tube. Colon contents were stored at −80 °C until further use. The remainder of the intestines was collected and prepared for histological analyses as previously described [17].

### 4.6. Bacterial Isolation and Next-Generation Sequencing

The Qiagen Power Soil Kit (Germantown, MD, USA) was used to isolate bacterial species within colon contents samples. Vanderbilt’s Microbiome Core sequenced the bacterial populations within each sample using 16s-based Next-Generation sequencing. Bacterial abundances were determined by visualizing and extrapolating sequence data from Krona Charts on the Illumina platform. Krona Chart data included the Shannon diversity index of each pup, as well as their relative abundances of various microbial populations. Extrapolated data from QIIME’s Krona Chart have previously been used in multidimensional analyses of the estimated relative abundances of microbes in a sample [34,35,36].

### 4.7. Assessment of Necrotizing Enterocolitis

Intestinal tissues were subjected to hematoxylin and eosin staining, and a histologic injury score was used to assess the development of Necrotizing Enterocolitis, as previously described [17]. A grade of 0–1 was considered healthy, whereas a grade of two or more was considered NEC; increasing scores indicate increased severity. Three blinded scorers were provided two images per pup and asked to determine an NEC score for each image and the scores were averaged. Three averaged scores for each pup were used to determine one NEC score per pup. 

### 4.8. Statistical Analysis

Group results were compared for significance using Prism’s one-way ANOVA and the Tukey post hoc test. For all microbial analyses, three non-littermates were used to obtain the average for each group. For histological analyses, 5–8 non-littermates were used per group. The presented images are representative of each group. The data are represented as the mean ± standard deviation and *p* < 0.05 was considered significant. Significance was determined by comparing each treatment group to F2_CT_ pups. In each group, approximately half of the pups were male and the other half were female. The majority of the pups were male in groups with uneven sample sizes. It should be noted that a major limitation of the current study was the small group sizes used for microbial analyses, due to the number of variables that were measured. 

## 5. Conclusions

Herein, we report that a paternal fish oil diet preconception influences early-life microbial seeding in the intestines of their offspring by increasing the relative abundance of Negativicutes. Pups with a paternal history of TCDD exposure were susceptible to NEC and this susceptibility was associated with an increase in the relative abundance of *Megashpaera*. Intervening with a paternal fish oil diet preconception significantly reduced the relative abundance of *Veillonella* and *Megasphaera*, but significantly increased the relative abundance of *Negativicoccus massiliensis*. This suggests that a paternal fish oil diet may reduce the incidence of NEC in pups with a history of toxicant exposure by influencing the relative abundances of *Veillonella*, *Megasphaera*, and *Negtivicoccus massiliensis* (types of Negativicutes) within the gut of their offspring. It should be noted that pups sired by unexposed fathers maintained on the standard diet exhibited a similar Firmicute diversity compared to pups susceptible to NEC, suggesting that factors other than gut dysbiosis, such as prematurity and inflammation, also drive disease development and outcomes. A limitation of this study was using an *n* of three for the Next Generation Sequence analyses due to the number of variables we assessed. Hence, this study should be repeated with a larger sample size and in other animal models of NEC to validate our current findings. 

## Figures and Tables

**Table 1:** Description of pup nomenclature used throughout the manuscript.

F2 Generation Nomenclature	Was the Pup’s Father (F1 Generation) Exposed to TCDD in Utero?	Did the Pup’s Father (F1 Generation) Receive a Fish Oil Preconception Diet?	Did the Pup (F2 Generation) Receive Postnatal Formula Supplementation?
F2_CT_	No	No	No
F2_CT_/Fish	No	Yes	No
F2_CT_/Form	No	No	Yes
F2_CT_/Fish/Form	No	Yes	Yes
F2_TCDD_	Yes	No	No
F2_TCDD_/Fish	Yes	Yes	No
F2_TCDD_/Form	Yes	No	Yes
F2_TCDD_/Fish/Form	Yes	Yes	Yes

**Table 2:** A paternal fish oil diet preconception reduces the incidence of NEC in offspring.

Pup Group	Average NEC Score	Overall NEC Incidence
F2_CT_	1.15	0/5 = 0%
F2_CT_/Fish	0.90	0/6 = 0%
F2_CT_/Form	1.7	2/5 = 40%
F2_CT/_Fish/Form	1.5	1/5 = 20%
F2_TCDD_	2.29	4/6 = 66%
F2_TCDD_/Fish	1.1	0/6 = 0%
F2_TCDD_/Form	2.22	5/6 = 83%
F2_TCDD_/Fish/Form	1.68	2/8 = 25%

**Table 3:** Patterns in Firmicute abundances among F2_CT_ and F2_TCDD_ pups based on their father’s preconception diet.

Paternal Standard Diet	Paternal Fish Oil Diet
↑ Bacilli	↓ Bacilli
↓ Negativicutes	↑ Negativicutes
↑ Selenomondales	↓ Selenomondales
↑ Veillonellaceae	↓Veillonellaceae
↓ Negativicoccus	↑ Negativicoccus
↑ Megasphaera	↓ Megasphaera
↑ Veillonella	↓ Veillonella

The arrows reference an increase or decrease in bacterial abundances in pups as it relates to their father’s preconception diet.

## Data Availability

All relevant data are within the manuscript.

## Supplementary Materials

The following supporting information can be downloaded at: https://www.mdpi.com/article/10.3390/md20060390/s1. Figure S1: A paternal fish oil diet preconception influences Negativicutes orders in intestines of F2_TCDD_ pups.
