# Supplementary material for: A Paternal Fish Oil Diet Preconception Modulates the Gut Microbiome and Attenuates Necrotizing Enterocolitis in Neonatal Mice
Source: Mar Drugs. 2022 Jun 13;20(6):390. doi: 10.3390/md20060390 (PMC9230221; doi:10.3390/md20060390)
Supplement: Supplementary file 1 [file marinedrugs-20-00390-s001.zip › marinedrugs-1758321-supplementary.pdf]

Supplementary Material

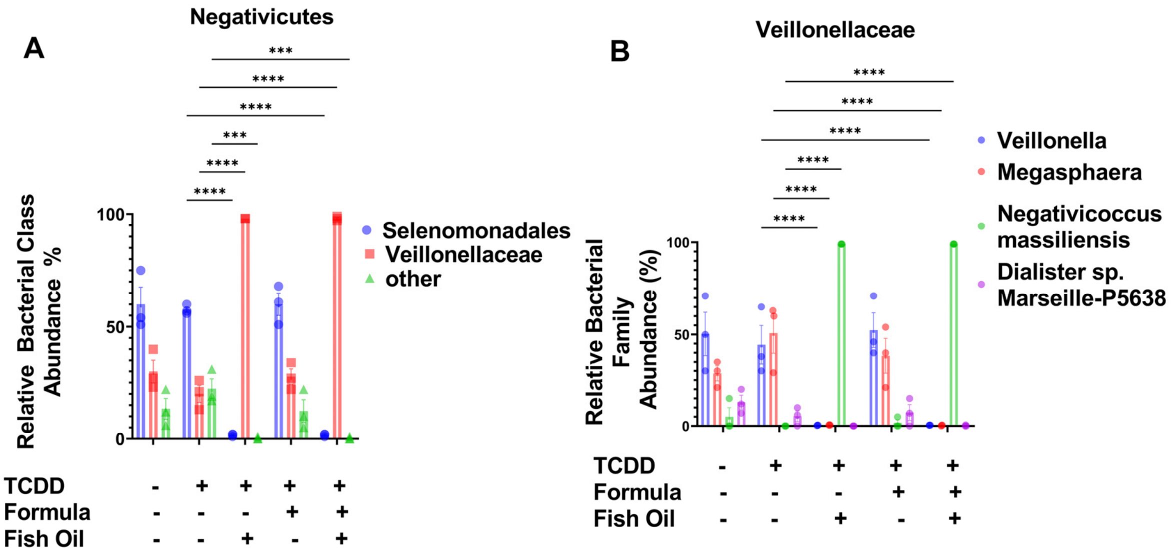

Figure S1: A paternal fish oil diet preconception influences Negativicutes orders in intestines of F2TCDD pups: (**A**) Bar graphs representing the relative abundance of Negativicutes, (**B**) *Veillonellaceae,* and (C) *Selenomondales,* abundances in the colon contents of offspring. Data were analyzed using one-way ANOVA and the Tukey Post Hoc Test; Data represent the mean +/− SD from 3 non-littermates *** $p \leq 0.001$, **** $p \leq 0.0001$.
